# Ectopic expression of a combination of 5 genes detects high risk forms of T-cell acute lymphoblastic leukemia

**DOI:** 10.1186/s12864-022-08688-1

**Published:** 2022-06-24

**Authors:** Li-Jun Peng, Yue-Bo Zhou, Mei Geng, Ekaterina Bourova-Flin, Florent Chuffart, Wei-Na Zhang, Tao Wang, Meng-Qing Gao, Meng-Ping Xi, Zhong-Yi Cheng, Jiao-Jiao Zhang, Yuan-Fang Liu, Bing Chen, Saadi Khochbin, Jin Wang, Sophie Rousseaux, Jian-Qing Mi

**Affiliations:** 1 Shanghai Institute of Hematology, State Key Laboratory of Medical Genomics, National Research Center for Translational Medicine at Shanghai, Ruijin Hospital Affiliated to Shanghai Jiao Tong University School of Medicine, Shanghai, China; 2 Laboratory of Molecular Pathology, Pôle de Recherches Sino-Français en Science du Vivant Et Génomique, Ruijin Hospital Affiliated to Shanghai Jiao Tong University School of Medicine, Shanghai, China; 3 Department of Oncology, Ruijin Hospital Affiliated to Shanghai Jiao Tong University School of Medicine, Shanghai, China; 4 UMR 5309, CNRSINSERM U1209Université Grenoble-Alpes/Institute for Advanced Biosciences, La Tronche, France; 5 Jingjie PTM Biolab (Hangzhou) Co., Ldt, Hangzhou, China

**Keywords:** T-ALL, Tissue-specific genes, Prognosis, Transcriptomic profile

## Abstract

**Background:**

T cell acute lymphoblastic leukemia (T-ALL) defines a group of hematological malignancies with heterogeneous aggressiveness and highly variable outcome, making therapeutic decisions a challenging task. We tried to discover new predictive model for T-ALL before treatment by using a specific pipeline designed to discover aberrantly active gene.

**Results:**

The expression of 18 genes was significantly associated with shorter survival, including ACTRT2, GOT1L1, SPATA45, TOPAZ1 and ZPBP (5-GEC), which were used as a basis to design a prognostic classifier for T-ALL patients. The molecular characterization of the 5-GEC positive T-ALL unveiled specific characteristics inherent to the most aggressive T leukemic cells, including a drastic shut-down of genes located on the mitochondrial genome and an upregulation of histone genes, the latter characterizing high risk forms in adult patients. These cases fail to respond to the induction treatment, since 5-GEC either predicted positive minimal residual disease (MRD) or a short-term relapse in MRD negative patients.

**Conclusion:**

Overall, our investigations led to the discovery of a homogenous group of leukemic cells with profound alterations of their biology. It also resulted in an accurate predictive tool that could significantly improve the management of T-ALL patients.

**Supplementary Information:**

The online version contains supplementary material available at 10.1186/s12864-022-08688-1.

## Background

T-cell acute lymphoblastic leukemia (T-ALL) emerges from a malignant monoclonal proliferation of cells that exhibit developmental arrest at varying stages of differentiation. Although modern intensified chemotherapy has greatly improved survival, long-term outcome of T-ALL adult patients remains unsatisfactory, with only 50% survival at 5 years [1, 2].

Currently, T-ALL treatment strategy largely relies on post-treatment minimal evaluation of residual disease (MRD) [3]. Assessment of MRD is usually carried out either by PCR amplification of clonotypic IG/TCR gene rearrangements or by flow cytometric detection of leukemia-associated phenotypes. MRD has been confirmed as a powerful predictor of long-term survival in adult patients with ALL in many studies [4–6]. However, MRD is not available at the time of diagnosis. In addition, a proportion of T-ALL patients diagnosed as MRD negative after the induction treatment will relapse. Therefore, there is still a need to find reliable biomarkers that could guide treatment or predict prognosis at diagnosis.

A deep understanding of the T-ALL pathogenesis, involving the expression of oncogenic transcription factors as well as genetic alterations, should contribute to the identification of relevant prognostic markers. So far, the expression of only a few transcriptional factors are currently used as predictive biomarkers or as indicators to help treatment planning [1, 7, 8]. Since NOTCH1 signaling plays a central role in T-cell lineage specification and NOTCH1 mutations have been found in up to 70% of adult T-ALLs, the relation-ship between gene alterations and prognosis has mainly focused on NOTCH1 signaling [9, 10]. A number of studies have also evaluated the prognostic relevance of the NOTCH1/FBXW7 mutation but it is still controversial [11–14]. Later, Trinquand and colleagues proposed to use the combination of NOTCH1/FBXW7 mutations and RAS and PTEN (NOTCH/FBXW7/RAS/PTEN) abnormalities as a refined oncogenetic classifier [12]. The NOTCH1/FBXW7/RAS/PTEN classification approach has not yet been evaluated in a Chinese population.

Our previous work demonstrated that malignant tumors frequently reactivate a large number of genes whose expression is normally tissue-restricted [15, 16]. There is emerging evidence that these aberrantly activated genes play pivotal roles in tumorigenesis and that they may serve as valuable cancer-specific biomarkers to predict prognosis as well as response to various treatments [17–21]. In particular, our investigations demonstrated that male germ cells express the largest number of tissue-restricted genes, and pointed to male-specific genes as a considerable reservoir of cancer biomarkers. Accordingly, we successfully identified the ectopic activation of a group of 26 male- and placental- specific genes as a predictor of poor prognosis in lung cancer [15]. Later, we found that the ectopic expression of six genes, which are normally expressed exclusively in embryonic stem cells, placenta or germ cells, could also predict prognosis in B cell acute lymphoblastic leukemia [22]. Altogether, our observations demonstrated that these ectopic expressions of tissue-restricted genes are potential source of new biomarkers to guide risk stratification and predict outcome [15, 22] as well as to help designing new therapeutic strategies [20, 23]. However, these ectopic expressions are highly context-dependent and the identification of the best relevant biomarkers requires an extensive analysis of their relationships and correlation with the clinical and biological data associated with each cancer type.

Here, we exploited genome-wide RNA sequencing of bone marrow samples obtained in a well characterized series of T-ALL patients, on which we applied our specifically designed strategy to detect the ectopic expression of tissue-restricted genes, and correlated these expressions with the survival probabilities of patients. This work led to the discovery of 18 genes, whose ectopic expression is significantly correlated with prognosis in T-ALL patients. By combining 5 of these genes, we defined an optimal classification system which, compared with a full assessment of the existing mutational status of NOTCH1/FBXW7/RAS/PTEN, largely improves our ability to predict outcome in T-ALL patients.

## Results

### The association of NFRP classes with event-free survival (EFS) is of borderline significance in our adult T-ALL patients

A total of 86 newly diagnosed adult T-cell acute lymphoblastic leukemia (T-ALL) were included in the present study (Table 1 and supp. Table S1). For 54 of these patients, RNA-seq data were available from our previous work [7]. The present study included an additional 32 patients, which without RNA-seq data but with detailed clinical information.

**Table 1:** Clinical characteristics and oncogenetic stratification of our training (RNA-seq data, *n* = 54) and test (RT-qPCR data, *n *= 32) cohorts of adult T-ALL patients

**Characteristics**	**Number of patients**	**N/F**		***p* value**	**NFRP**		***p* value**
		**Mutation**	**WT**		**class I**	**class II**	
**Total**	86	67	19	/	53	33	/
**Gender, male/female**							
Male	58	47	11	0.406	36	22	1.0
Female	28	20	8		17	11	
**Age, years, > 35y**							
< 35	53	43	10	0.427	36	17	0.172
≥ 35	33	24	9		17	16	
**WBC, × 10**^**9**^**/L**							
≤ 100	68	52	16	0.751	43	25	0.593
> 100	18	15	3		10	8	
**Immunophenotype**							
ETP	27	20	7	0.584	12	15	0.033
non-ETP	59	47	12		41	18	
**Karyotype**							
Normal karyotype	43	33	10	0.215	25	18	0.218
Abnormal karyotype	31	28	3		23	8	
**CR**							
Yes	61	49	12	0.405	38	23	1.0
No	25	18	7		15	10	
**MRD-Induction**							
≥ 10^4	45	33	12	0.290	24	21	0.117
< 10^4	38	32	6		27	11	

NFRP class I: positive for NOTCH1/FBXW7 (N/F) and negative for RAS and PTEN mutations (*n* = 53); NFRP class II: other mutational status (negative for NOTCH1/FBW7 (N/F) and/or positive for RAS and PTEN mutations) (*n* = 33). EFS, Event-free survival; WBC, White Blood Cells counts; ETP, Early T cell Precursors; CR, complete remission; MRD, Minimal Residual Disease; *p*-value determined by using two-sided Fisher's exact test

The N/F mutational status as well as the N/F combined with RAS and PTEN (NFRP) mutation status were reported to impact adult T-ALL patients [11–14]. Therefore, NOTCH1, FBXW7, RAS and PTEN mutation status were also assessed for all our T-ALL adult patients. Clinical and biological features of patients with T-ALL were analyzed according to the mutational status of N/F or NOTCH1/FBXW7/RAS/PTEN (NFRP classes) as summarized in Supplementary Table S1. NFRP classes were defined as follows: patients with N/F mutation but without RAS or PTEN mutations were assigned to class I, and the patients with other mutational status were assigned to class II as defined by Trinquand et al. [12]. There was no significant association between oncogenetic classifiers and clinical features. Noticeably, early T-cell precursor (ETP) ALL was more frequently observed in NFRP class II than in NFRP class I (45.5% versus 22.6%; *p* = 0.033).

We then analyzed the impact of N/F mutational status and NFRP classes on patient survival probabilities considering overall survival (OS) and event-free survival (EFS). N/F mutated patients showed increased OS and EFS, although for OS it was of borderline significance (with log-rank *p* = 0.049 for OS and *p* = 0.01 for EFS) (Supplementary Fig. S1 A). Consistent with Trinquand and colleagues [12], prognostic prediction ability of NFRP classes was improved compared to the classification based only on the N/F mutational status. Indeed, NFRP class II patients predicted significantly shorter OS and EFS than those of NFRP class I (log-rank *p* = 0.037 for OS and *p* = 0.009 for EFS) (Supplementary Fig. S1B). However, the NFRP classifier only remained a significant prognostic covariate for EFS when adjusting to age (using the 35-year cutoff) and WBC count (using the 100 × 10^9/L cutoff) (EFS: HR = 1.751; 95% CI, 1.011 to 3.03; *p* = 0.045; and OS: HR = 1.623; 95% CI, 0.917 to 2.873; *p* = 0.097).

### A combination of ectopically expressed genes can be used to reliably predict prognosis of T-ALL patients at diagnosis

These observations prompted us to seek for new biomarkers which could reliably stratify patients before treatment.

We applied a strategy specifically designed to identify the aberrant expression of genes which are normally silent in non-germline adult tissues and to test the association of these ectopic expressions with survival probabilities.

By using available RNA-seq data in large series of normal human tissues, we identified 3195 transcripts with an expression restricted to testis, placenta or embryonic stem cells, of which 448 were found ectopically expressed in at least 10% and not in more than 90% T-ALLs samples. We then used a first cohort of T-ALL patients for whom RNA-seq as well as survival data were available. In addition to the 54 T-ALL adult patients, in order to strengthen the power of the approach, RNA-seq data obtained from 55 samples of children with T-ALL were also included in the training cohort (described in Supp. Table S1 ). Since our main objective was to identify a common molecular background related to aggressiveness in both children and adult T-ALL, our approach was designed to identify a subset of genes whose expression is associated with poor prognosis considering either the whole population of T-ALL, including children and adult patients, or sub-groups of children or adult patients. Considering each of the 448 genes ectopically expressed in a subgroup of T-ALL, we compared survival probabilities of the two groups of patients, whose malignant cells respectively did or did not express the gene. A total of 18 different genes (listed in Supplementary Table S2) were identified whose activation was significantly associated with OS and/or EFS in our T-ALL series. The individual association between the expression of each of the 18 genes and survival is shown is Supp. Fig. S2. The relative importance of each gene for risk stratification was also evaluated by a multivariate Cox model (Supp. Table S3).

**Fig. 1:**
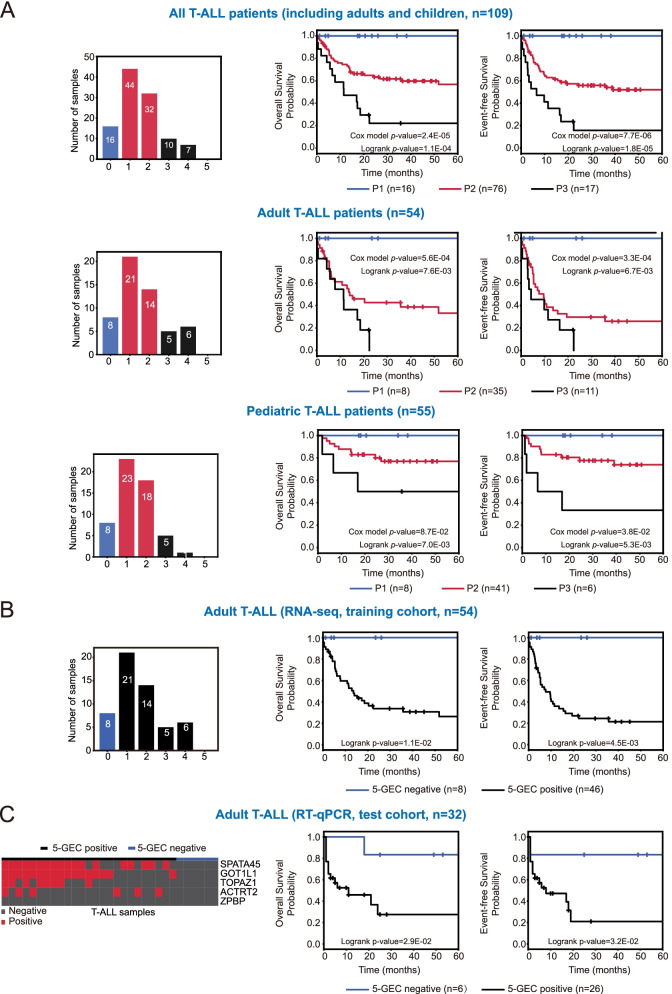
Stratification of T-ALL patients with the combination of 5 ectopically expressed genes (5-GEC). **A** Left panels: bar plots showing the distribution of the number of positive genes of the 5 ectopically expressed genes in all the T-ALL patients from our training cohort (including adults and children) (upper panel), or considering either adult T-ALL patients (middle panel) or children T-ALL patients (lower panel). Prognostic groups were defined according to the number of positive genes as follows: P1 (blue) = none of the 5 genes are activated, P2 (red) = 1 or 2 genes are positive, P3 (black) = 3 to 5 genes are activated. Center and right panels: Kaplan-Meier survival curves comparing overall survival (OS, center panel) and event-free survival (EFS, right panel) between the P1, P2 and P3 groups (as determined above, same color code), in the same three subsets of patients (respectively upper, middle and lower panels). The Cox model and logrank *p*-values are shown for each plot. *P*-values obtained from univariate Cox's proportional hazards model reflects how well the number of expressed ectopic genes can explain the survival. Logrank *p*-value reflects the difference between the survival probabilities in P1, P2 and P3 prognosis groups. **B **and** C** 5-GEC positivity associates with shorter survival in adult T-ALL patients training and test cohorts. **B** Training cohort (*n* = 54, adult T-ALL samples analyzed by RNA-seq, same as in A middle panels). Left panel: bar plot showing the proportion of patients according to the number of positive 5-GEC genes; Center and right panels: Kaplan-Meier plots showing overall survival (OS, center panel) and event-free survival (EFS, right panel) between 5-GEC negative (none of the 5-GEC are expressed) and positive patients (at least one of the 5-GEC expressed). **C** Test cohort (*n* = 32, samples analyzed by RT-qPCR). Left panel: Heatmap showing positive samples for the 5-GEC genes; Center and right panels: Kaplan-Meier plots illustrating overall survival (OS, center panel) and event-free survival (EFS, right panel) between 5-GEC negative and positive patients

In order to assess the value of combinations of these genes in terms of prognostic biomarkers, we then tested all possible combinations of the 18 genes for their potentiality to stratify T-ALL patients, as detailed in Supp. Methods and Supp. Fig. S3. Among them, the *5*-gene set of ZPBP, GOT1L1, ACTRT2, SPATA45 and TOPAZ1 (all restricted to male germ cells) was identified as an optimal classifier for prognostic stratification in T-ALL patients (*p* < 10^–4^ for OS and *p* < 10^–5^ for EFS). As illustrated by Kaplan–Meier plots in Fig. 1A, a stratification of patients by the number of positive expressions of the 5 genes can well separate patients into different risk groups considering all T-ALL cases (upper panels), or subsets of either adult (middle panels) or pediatric (lower panels) T-ALL patients. All T-ALL patients were then assigned to 2 groups according to the ectopic activation of the 5 genes (Fig. 1B). Those expressing at least one of the 5 genes were assigned to the “5-gene expression classifier” (5-GEC) positive group. The other patients, expressing none of the five genes were assigned to the 5-GEC negative group. In particular, 5-GEC positive and negative T-ALL adult patients showed significant differences in terms of survival probabilities (log-rank *p* = 0.01 for OS and *p* = 0.004 for EFS (Fig. 1B)). In addition, a multivariate survival Cox model including age as an explanatory variable along with the 5-GEC classifier demonstrates that the 5-GEC classifier remains significantly associated with survival even when age is taken into account (Supp. Table S4).

In order to validate the predictability of the 5-GEC, we detected the expression of the 5 genes in a second cohort, the test cohort of 32 T-ALL adult patients by using RT-qPCR. As a result, out of the 32 cases, 6 patients were assigned to the 5-GEC negative group, whereas the other 26 patients were 5-GEC positive. Kaplan–Meier plots also demonstrated significant differences in both OS and EFS (Log-rank test *p* = 0.029 and *p* = 0.032 respectively, Fig. 1C).

### A stratification based on 5-GEC predicts MRD status and identifies MRD negative patients with high risk of relapse

**Fig. 2:**
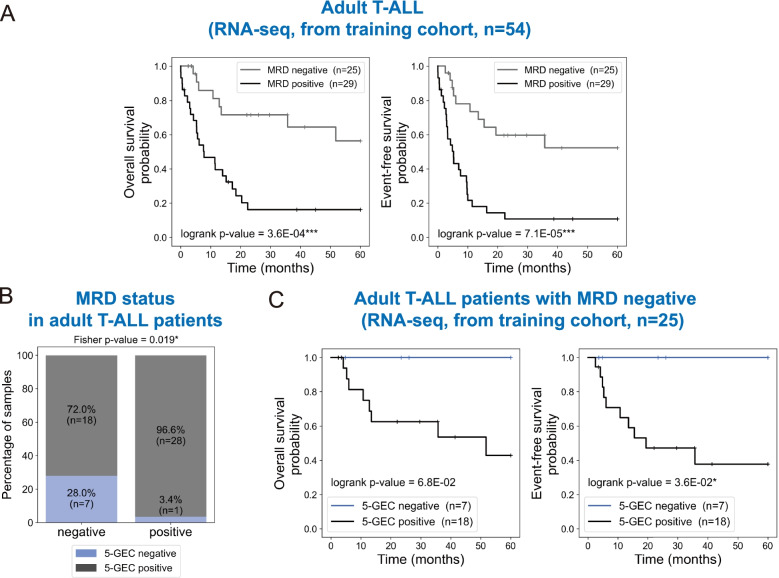
A 5-GEC positive signature predicts high risk of later relapse in MRD negative adult patients. **A** Kaplan–Meier plots of overall survival (OS, left) and event-free survival (EFS, right) in MRD negative and MRD positive patients (adult T-ALL, *n* = 54)**.** **B** Distribution of 5-GEC negative and 5-GEC positive samples according to MRD status in adult T-ALL patients. The MRD positive group is significantly enriched in 5-GEC positive samples compared to the MRD negative group. The corresponding *p*-value of the Fisher’s exact test = 0.019. **C** Kaplan–Meier plots showing overall survival (OS, left) and event-free survival (EFS, right) in 5-GEC positive and negative patients amongst the MRD negative adult patients’ subgroup

MRD status following induction therapy in patients with ALL has been routinely used to predict outcome, and has been reported to strongly and consistently associate with clinical outcomes in ALL [4]. Consistently, positive MRD was predictive of significantly inferior OS and EFS in our cohort (*p* < 0.001 for both OS and EFS, Fig. 2A). However, MRD status is not available at the time of diagnosis. Additionally, recurrence of the disease also occurs in patients with negative MRD decreasing the probability of overall and event-free survival. Interestingly, our newly identified 5-GEC classifier turned out to also be an efficient predictor of MRD positivity (Fig. 2B, Fisher test, *p* = 0.019). Moreover, within the MRD negative subgroup, 5-GEC positivity was also significantly associated with shorter survival (*p* = 0.036 for EFS, Fig. 2C), thus differentiating patients who are likely to respond well to standard therapy from those who may benefit from more intensive therapy. These observations were further confirmed in our test cohort (Supplementary Fig. S4).

### Gene expression profile of 5-GEC positive T-ALL is significantly depleted in genes involved in basic cellular activities and identifies specific characteristics in MRD negative / 5-GEC positive T-ALL.

**Fig. 3:**
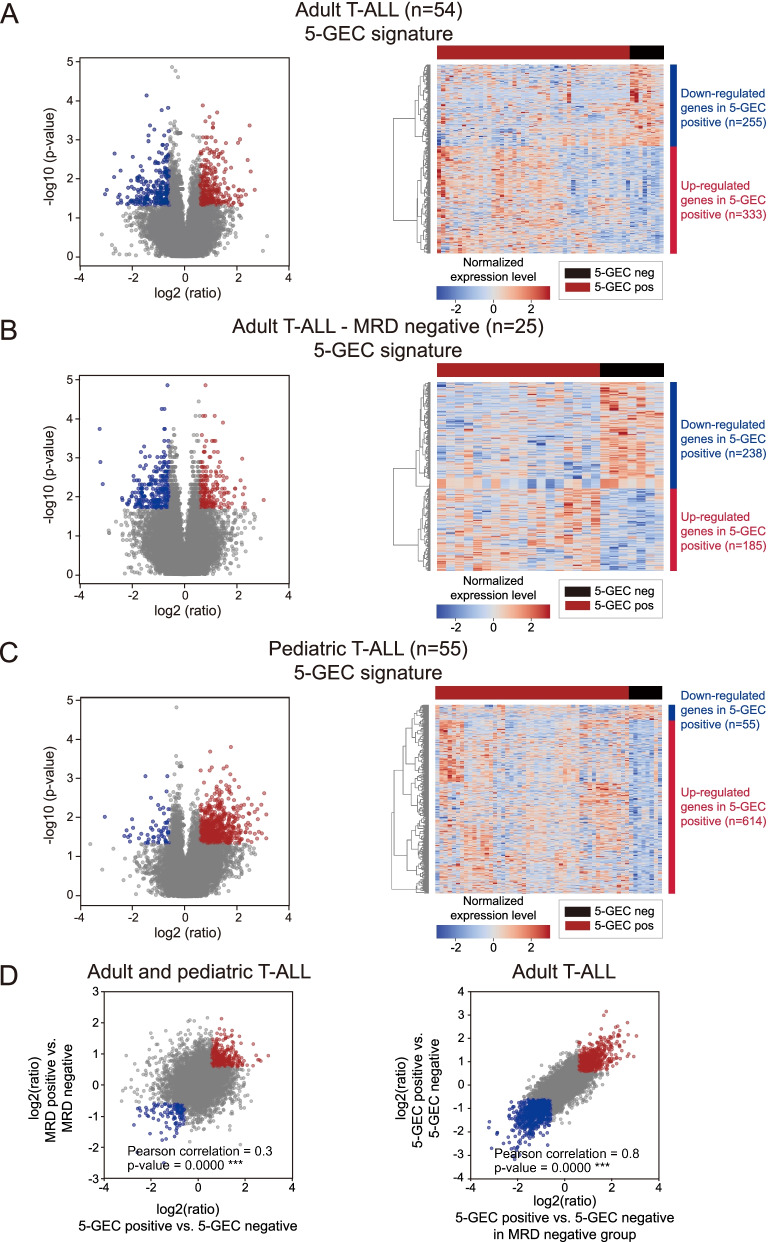
Differential expression profiles of 5-GEC positive versus 5-GEC negative subgroup.** A-C** Differential expression profiles of 5-GEC positive aggressive T-ALL in adult patients (**A** and **B**) and children (**C**) of the training dataset. The volcano plots (left panels) and heatmaps (right panels) illustrate the transcriptomic profile of 5-GEC positive versus negative T-ALL in all adult patients of the training set (**A**, *n* = 54), in adult patients with MRD negative status (**B**, *n* = 25) or in children (**C**, *n* = 55). The *p*-values used in volcano plots were calculated with the Wilcoxon statistical test. The differentially expressed genes used for the heatmaps were selected with a *p*-value < 0.05 and abs (ratio) > 1.5. In all heatmaps the genes and samples were clustered using Euclidian-based distance hierarchical clustering with Ward’s linkage; **D** Correlation plots comparing gene expression signatures. Left panel: correlation plot comparing the gene expression signature of 5-GEC positive versus negative patients (x axis) with the signature of MRD positive versus negative patients (y axis) in all T-ALL patients of the training cohort (*n* = 109). Right panel: correlation plot comparing the gene expression signatures of 5-GEC positive versus negative patients considering adult T-ALL patients (*n* = 54, y axis) or considering adult T-ALL patients with MRD negative status only (*n* = 25, x axis)

**Fig. 4:**
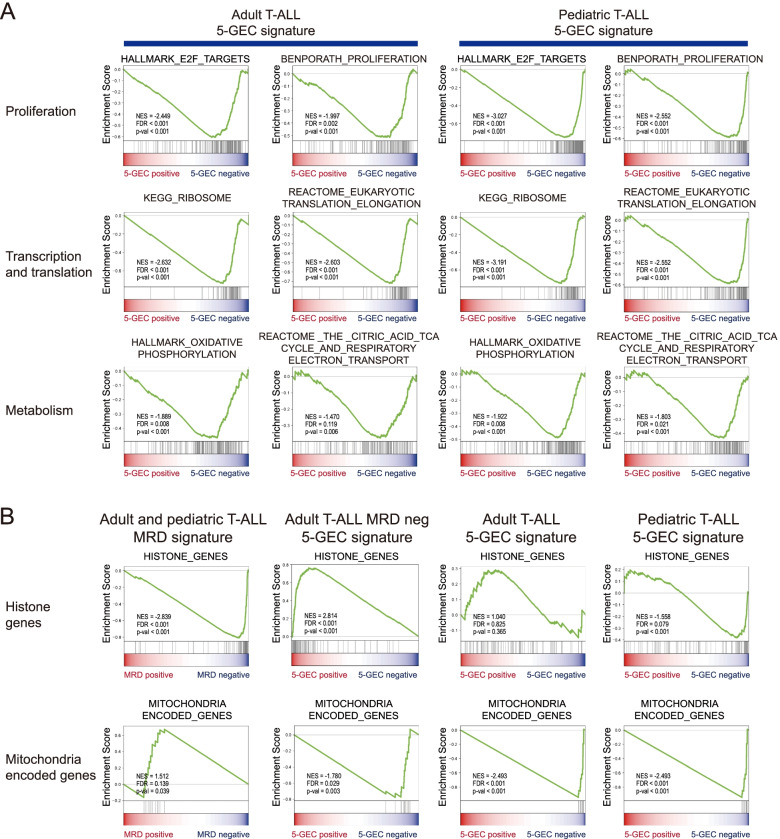
Gene Set Enrichment Analysis (GSEA) shows specific features of 5-GEC positive T-ALL.** A **and** B **Gene Set Enrichment Analysis (GSEA) shows specific features of 5-GEC positive T-ALL in adult patients (*n* = 54, left panels) or in children (*n* = 55, right panels) **A** GSEA plots illustrating genesets corresponding to the most down-regulated functions/signatures in the 5-GEC positive ALL. **B** Hematopoietic stem cells genes are down regulated in 5-GEC positive adult T-ALL signature. **C** Comparison of the transcriptomic signatures between MRD positive T-ALL and 5-GEC positive T-ALL. GSEA plots that illustrating different enrichment/depletion profiles in MRD positive T-ALL (1^st^ column) compared to 5-GEC positive signatures in MRD negative T-ALL adult patients (2^nd^ column) or in all T-ALL adult patients (3^rd^ column), or in T-ALL children patients (4^th^ column). For all the genesets, the depletion was significant with a nominal *p* < 0.01 and FDR < 0.2. The geneset “histone genes” corresponds to the human histone encoding genes identified in El Kennani et al. 2018 ([24]). The geneset “mitochondria encoded genes” is constituted of the 13 mitochondrial genes of the human genome. neg: negative; pos: positive. All other genesets were selected from the MSIG database of the Broad Institute (categories C2, C5, C7 or H of the MsigDB). neg: negative; pos: positive

Differential expression analyses (Fig. 3 and Supp. Fig. S5) and the corresponding GSEA (Fig. 4, Supp. Fig S6 and S7, and Supp. Table S5) were performed for the five following experimental designs including i/ 5-GEC signature (positive versus negative) in all T-ALL (pediatric and adult, *n* = 109), ii/ 5-GEC signature in adult T-ALL (adult samples of the training cohort, *n* = 54), iii/ 5-GEC signature in adult T-ALL with MRD negative status (subset of the adult training cohort, *n* = 25), iv/ 5-GEC signature in pediatric T-ALL (children of the training cohort, *n* = 55), v/ MRD signature (positive versus negative) in all T-ALL (pediatric and adult samples of the training cohort for whom the MRD status is available, *n* = 103).

The volcano plots and heatmaps shown Fig. 3 illustrate the expression of the genes down- and up-regulated in 5-GEC positive versus negative patients T-ALL patients with an absolute fold change of expression values between 5-GEC positive and negative patients above 1.5 and a Wilcoxon statistical test *p*-value < 0.05. Respectively 255 and 333 genes were down and up regulated considering all adult T-ALL patients (Fig. 3A), and respectively 238 and 185 genes were down and up regulated considering only MRD negative adult T-ALL patients (Fig. 3B). In pediatric T-ALL, respectively 55 and 614 genes were down and up-regulated in 5-GEC positive compared to 5-GEC negative cases (Fig. 3C).

In order to characterize the molecular profile of 5-GEC positive aggressive T-ALL we performed Gene Set Enrichment Analysis (GSEA) to highlight biological pathways correlating with that of 5-GEC positive versus negative T-ALL samples.

Interestingly, the GSEA profiles of these aggressive forms of T-ALL revealed a major down-regulation of most cellular activities. Gene sets constituted of genes involved in cell proliferation and mitosis, or RNA ribosomal and translation activities, as well as mitochondria and related metabolic activities, were among the most significantly downregulated in 5-GEC positive T-ALL (Fig. 4A), suggesting that these aggressive T-ALL forms were those enriched in “dormant” cells. Remarkably, the 5-GEC positive adult T-ALL cells are not expressing many of the genes normally expressed in hematopoietic stem cells (Fig. 4B). GSEA also shows that most genesets and pathways are depleted or enriched in both adult and children 5-GEC positive T-ALL (Fig. 4A, Supp. Fig. S6 and S7), highlighting similarities in the transcriptomic signatures associated with aggressiveness in the two populations. Interestingly the same observation came out from our previous study in B-ALL, where many similarities in the global transcriptomic signatures associated with aggressiveness were shared between adult or children B-ALL [22] despite the reported differences between the two contexts. However, in the case of T-ALL, several genesets, were found differentially enriched/depleted in adult patients or children, including hematopoietic stem cells genes, downregulated in 5GEC positive adult T-ALL and upregulated in pediatric T-ALL (Fig. 4B). 

Interestingly, part of the transcriptomic profile of 5-GEC positive T-ALL is also shared with MRD positive T-ALL. Indeed, 5-GEC positive ALL and MRD positive ALL were both depleted for gene sets representative of genes involved in cell proliferation, E2F and MYC targets, ribosome biogenesis and translational processes, as well as oxidative phosphorylation (Supp. Fig. S6 and S7).

However, genes differentially expressed between 5-GEC positive and negative adult T-ALL samples only partially overlap with those differentially expressed between the MRD positive or negative subgroups. Indeed, the gene expression signature of 5-GEC positive versus negative patients considering all T-ALL adult patients (*n* = 54) and the gene expression signature of MRD positive versus negative patients are weakly correlated (Pearson coefficient = 0.3), while the gene expression signatures of 5-GEC positive versus negative patients considering all T-ALL adult patients (*n* = 54) or considering T-ALL patients with MRD negative status only (*n* = 25) are highly correlated (Pearson coefficient = 0.81) (Fig. 3D). This suggests that 5-GEC positive T-ALL had specific characteristics that may explain why some of them which were detected as MRD negative were actually still prone to relapse. Indeed, several pathways and functions are specifically associated with the 5-GEC signature and not shared by MRD positive ALL.

The GSEA signature of 5-GEC positive ALL within the MRD negative group well illustrates this specificity. One specific feature of the 5-GEC positive MRD negative T-ALL signature is that it is highly enriched in mRNAs from genes encoding histones and chromatin proteins as opposed to MRD positive T-ALL, which show a depletion for these same mRNAs (Fig. 4C and Supp. Fig S6 and S7). Another striking characteristic of 5-GEC positive T-ALL is the complete shutdown of mitochondria-encoded transcripts. Indeed, mitochondria related genes are globally depleted in both MRD positive and 5-GEC positive cells, but the expression of the 13 genes located on the mitochondria genome remain high in MRD positive T-ALL (as compared to MRD negative). In 5-GEC positive T-ALL, the situation is different since these same 13 genes are completely shut down (Fig. 4C and Supp. Fig S6 and S7), suggesting that a dramatic impairment of mitochondria transcriptional activity is specifically associated with these 5-GEC positive T-ALL.

## Discussion

In this study, we found that patients positive for N/F mutation only have a trend towards a more favorable outcome, whereas NFRP class I was significantly correlated with longer survival, in agreement with data reported by the GRAALL group [12]. However, this oncogenetic classifier based on NFRP classes only remained of marginal significance for the prediction of OS and EFS in the multivariate analysis.

Based on our previous work, we stratified patients according to the aberrant/ectopic expression of genes that are normally epigenetically repressed in most non-tumor adult somatic cell types. We found that a combination of a subset of 5 tissue-restricted genes (5-GEC) could efficiently stratify patients into groups with different prognosis. In addition, this new classification system could also predict prognostic in an independent group of patients. More importantly, this new classification system implemented at the time of diagnosis could predict MRD positivity with high efficiency, since nearly all MRD positive patients had been assigned to the 5-GEC positive group. Additionally, MRD negative patients which had been assigned to the 5-GEC negative group showed no event of relapse or death, whereas the MRD negative patients of the 5-GEC positive group were of significantly higher risk of death or relapse.

In particular, we adapted our approach to fully exploit RNA-seq data, which provide a more accurate and efficient technology to explore transcriptomes. This enabled the detection not only of ectopically activated protein-coding genes but also of tissue-specific non-coding sequences. Our results here suggest that these non-coding transcripts actually largely contribute to ectopic activations. Indeed, among the 18 genes whose expression was associated with inferior survival in T-ALL, 11 were protein-coding genes, whereas 7 corresponded to non-coding sequences. The roles and functions of these non-coding transcribed RNAs in their normal context of expression or in cancer cells are entirely unknown and their discovery opens a new field for future research.

The normal functions of the protein-coding genes themselves are also poorly known. Among them, GOT1L1 was reported to show L-aspartate aminotransferase activity and thus could be involved in the synthesis of D-aspartate, which serves as the agonist of N-methyl-D-aspartate receptor (NMDAR) [25]. Leanne et al. [26] reported that low activity of NMDAR is significantly correlated with favorable patient prognosis in several cancer types, which may provide a possible explanation to our finding that high expression of GOT1L1 is associated with shorter survival and a mechanism of GOT1L1 in leukemogenesis. TOPAZ1 contains an evolutionarily conserved domain named PAZ, which is involved in the specific recognition of siRNAs [27]. It has been suggested that the PAZ do-main plays an important role in regulating human embryonic stem cell and glioma stem cells self-renewal [28, 29]. These observations suggest potential mechanisms by which these genes could contribute to cancer development, but detailed investigations are required to fully understand their functions and the impact of their ectopic expression in cancer cells. Although the biological roles and functions of these five testis-specific genes remain to be discovered, the fact that their expression is restricted to male germ cells and cancer, makes them as very attractive therapeutic targets.

We also found that the aggressive 5-GEC T-ALL were mostly depleted in pathways that are essentially involved in active and proliferative cells, such as RNA and DNA synthesis, mitosis and DNA replication. Interestingly, these pathways were also downregulated in MRD positive as compared to MRD negative T-ALL. Based on recent reports that ribosome and protein biogenesis function in normal and leukemic stem cells [30–32], it is reasonable to speculate that these changes might be associated with metabolic changes and involved in T-ALL progression and treatment resistance. Moreover, E2F and MYC target genes were among the most depleted gene sets in both MRD positive and 5-GEC positive groups. These findings further reinforce the overwhelming importance of the proliferative status in the ability of cells to respond to chemotherapy in cancer [33, 34]. Additionally, the RB-E2F pathway is also known to play a pivotal role in cell proliferation [35–37] and has recently been reported to play a critical role in controlling cell quiescence depth [38]. These reports suggest that MRD negative or 5-GEC negative ALL patients would be more likely in a state of hyper-proliferation, and therefore prone to respond more efficiently to chemotherapy. This is also consistent with results from pediatric B-ALLs which showed that under-expression of genes promoting cell proliferation is associated with resistance to chemotherapy [39].

Although there are many common features in the expression profiles shared between MRD positive and 5-GEC positive T-ALLs, the latter still has its unique features. Our 5-GEC positive group has a higher contingent of “dormant" cells that show extremely low translation, transcription and proliferation rates and low mitochondrial activity. Most strikingly, genes located on the mitochondrial genome are totally silenced in 5-GEC positive T-ALL, whereas they are still expressed in the MRD positive T-ALL. On the basis of recent reports that mitochondrial and metabolic remodeling is a central feature of normal and leukemic stem cells [31, 40] and that regulated mitochondrial metabolism is required to maintain stem cell self-renewal [41], our results further strengthen the notion that mitochondrial dormancy is an important characteristic of stem cells and could be involved in chemotherapy resistance and disease progression. However, although the transcriptomic signature of 5-GEC positive leukemia suggests a “dormant” phenotype, we have no additional evidence for a “stem cell-like” nature of the 5-GEC positive leukemic cells. Actually, as illustrated in Fig. 4B, the 5-GEC positive T-ALL signature in adult patients is depleted in hematopoietic stem cell expression signatures, although it is enriched in 5-GEC positive pediatric T-ALL. Thus, our new gene expression classifier is more likely to link prognosis with the pathogenesis of a specific form of aggressive T-ALL and may provide a lead to better explain malignant transformation and progression of these ALL.

## Conclusions

T cell acute lymphoblastic leukemia (T-ALL) is an aggressive hematologic disease associated with dismal survival in adult patients. Despite extensive exploration of the genetic and epigenetic landscapes of T-ALL, prognostic biomarkers that could guide treatment selection mostly rely on post-induction minimal residual disease. Identification of novel biomarkers that can stratify patients at diagnosis is still needed. Following a dedicated strategy to screen whole genome expression data in T-ALL samples, Peng et al. scored the out-of-context activation of silent tissue-restricted genes. By correlating these expressions with survival probabilities, they identified a set of 5 genes, whose awakening not only predicted positive minimal residual disease but also a high risk of relapse in a subset of patients with apparently negative minimal residual disease. The 5-genes positive T-ALL also pointed to a particular metabolic state of the aggressive T-ALL group harboring a low mitochondrial genome activity.

## Methods

### The patients and samples

From 2009 to 2018, 86 T-ALL adult patients, aged from 15 to 67 years, were treated in the Shanghai Institute of Hematology (SIH)-based hospital network or Multicenter Hematology-Oncology Protocols Evaluation System (M-HOPES) in China. Patients were all enrolled in an SIH protocol [Chinese Clinical Trial Registry, number ChiCTR-RNC-14004969 (for sample collection) and ChiCTR-ONRC-14004968 (for treatment)] as previously described [42]. All patients provided informed consent and for patients below age 16 guardians provided informed consent for sample collection and research in accord with the Declaration of Helsinki.

Genomic DNA and total RNA of bone marrow were extracted using AllPrep DNA/RNA/Protein Mini Kit (Qiagen) or TRIzol reagent (Invitrogen). Bone marrow minimal residual disease (MRD) was analyzed by flow cytometry at the end of the induction treatment. MRD negative was defined as < 0.01% residual leukemia cells. MRD was not available in 3 patients, who all died before the end of induction treatment.

### Gene expression datasets

RNA-Seq data from 13 normal samples were used as control from the dataset PRJEB4337 available on NCBI BioProject portal (https://www.ncbi.nlm.nih.gov/bioproject/?term=PRJEB4337) including 4 bone marrow samples (SAMEA2162823, SAMEA2149004, SAMEA2154529, SAMEA2163105), 5 lymph node samples (SAMEA2149876, SAMEA2150385, SAMEA1965299, SAMEA2155628, SAMEA2152719) and 4 spleen samples (SAMEA2153031, SAMEA2155751, SAMEA2159764, SAMEA2146236).

### RNA-Seq data analysis

Raw RNA-seq data obtained from bone marrow samples of 109 pediatric (*n* = 55) and adult T-ALL (*n* = 54) enrolled in our center [7] as well as from 13 normal samples from the dataset PRJEB4337 available on NCBI BioProject portal (https://www.ncbi.nlm.nih.gov/bioproject/?term=PRJEB4337) were used for the detection of aberrant expression of genes and correlation with prognosis. Reads from fastq files were aligned using STAR 2.5.2b software for UCSC hg19 reference genome. The aligned reads were counted using HTSeq framework (version 0.9.1). RPKM (reads per kilobase million) values were obtained by dividing the RPM (reads per million) values by a cumulated length of exons in kilobases and log-transformed by computing log2(1 + RPKM).

### Analysis of mutational profiles

Mutation calling from RNA-seq data of training cohort has been reported previously [7]. Mutational hotspot regions of NOTCH1, FBXW7, PTEN, NRAS and KRAS were sequenced using Sanger sequencing in the 32 additional patients of the test cohort. The primer sequences used for NOTCH1 and PTEN were the same as previously described [43, 44].

### Identification of biomarkers of aggressive T-ALL based on ectopic expression of tissue-specific genes

A dedicated bioinformatic pipeline was applied first to identify genes with tissue-specific expression and second to detect their aberrant expression in T-ALL. Using RNA-Seq expression data from different normal human tissues, we first identified 3195 transcripts whose expression was restricted to testis, placenta or embryonic stem cells. None of these genes are expressed in normal hematopoietic tissues. Second, for each tissue-restricted gene, we established a threshold of log-transformed RPKM values differentiating background noise from expression, and then compared the expression value of each T-ALL sample with the threshold. The expression data in T-ALL samples were binarized, positive if the expression value was above the threshold, and negative otherwise. Procedures of these two steps are listed in the Supplementary methods.

### Analysis of association between ectopic expression and patient outcome

Cox proportional hazard model was used in order to test if the expression of the gene was significantly associated with overall survival (OS) and event-free survival (EFS). The ectopic expression of a gene was considered as significantly associated with the survival if the Cox model *p*-value was less than 0.05 and the hazard ratio above 1.5. The statistics and bioinformatic pipelines for survival analysis and the design of optimal combinations of genes are detailed in the Supplementary methods.

### Real-time qualitative PCR (RT-qPCR) test of the aberrant expression of the 5 ectopic genes

cDNA was synthesized from total RNA using Super-Script III First-Strand Synthesis SuperMix Kit (Invitrogen) according to the manufacturer’s procedures. RT-qPCR reactions using SYBR Green (TaKaRa) and a 7500 ABI RT-qPCR machine (Applied Biosystems, USA). The 2^−ΔΔCt^ method was used to estimate the fold induction of each gene as described in Rousseaux *et.al* [15]. In short, the expression value was calculated (2^(Ct of gene of interest in testis – Ct of gene of interest in sample))/ (2^(mean Ct of the 4 control genes in testis – mean Ct of the 4 control genes in sample)), and expressed as the ratio of expression relative to testis. The four control genes were *Actin, U6, RELA, AUP1*. Assays were done in triplicates. Seven normal bone marrow samples and three cord-blood samples were used to determine a threshold of aberrant expression (corresponding to the mean expression value + two standard deviations of these 10 samples). A gene was considered positively expressed when its expression value was found above this threshold.

### Gene Set Enrichment Analysis (GSEA)

GSEA (https://www.gsea-msigdb.org/gsea/index.jsp,) [45, 46] was carried out on the collections of gene sets made available by the Broad Institute (MSigB: http://software.broadinstitute.org/gsea/msigdb/index.jsp) using the GSEA software available on the website.

### Statistical analysis

Fisher's exact tests were used to compare categorical variables. Overall survival (OS) and event-free survival (EFS) were measured from the date of diagnosis of T-ALL to the date of death (OS and EFS) or relapse (EFS) or to the date of last contact (censored). Log-rank test was used to compare OS or EFS survival between groups and illustrated by Kaplan–Meier curves. The last follow-up was carried out in September 2020. Multivariate analyses were performed using Cox proportional hazard models. *P*-values < 0.05 were considered statistically significant. We used open source packages available in R (version 3.3.0) and Python (version 3.7, packages scipy and lifelines) to perform statistical analyses.

## Supplementary Information

**Additional file 1.**

**Additional file 2.**

## Data Availability

The dataset has been deposited at The National Omics Data Encyclopedia (NODE) (https://www.biosino.org/node), under accession no. OEP000760 or the following URL: https://www.biosino.org/node/project/detail/OEP000760.

## Abbreviations

T-ALL: T cell acute lymphoblastic leukemia
MRD: Minimal residual disease
RPKM: Reads per kilobase million
OS: Overall survival
EFS: Event-free survival
RT-qPCR: Real-time qualitative PCR
GSEA: Gene Set Enrichment Analysis
RNA-seq: RNA sequencing
N/F: NOTCH1/FBXW7
NFRP: NOTCH1/FBXW7 combined with RAS and PTEN status
ETP-ALL: Early T-cell precursor ALL
HR: Hazard ratio
CI: Confidence intervals
5-GEC: 5-Gene expression classifier
NMDAR: N-methyl-D-aspartate receptor

## References

[1] Marks DI, Rowntree C (2017). Management of adults with T-cell lymphoblastic leukemia. Blood.

[2] Jabbour E, Pui CH, Kantarjian H (2018). Progress and Innovations in the Management of Adult Acute Lymphoblastic Leukemia. JAMA Oncol.

[3] O'Connor D (2018). Refining genetic stratification in T-ALL. Blood.

[4] Berry DA, Zhou S, Higley H, Mukundan L, Fu S, Reaman GH, et al. Association of Minimal Residual Disease With Clinical Outcome in Pediatric and Adult Acute Lymphoblastic Leukemia: A Meta-analysis. JAMA Oncol. 2017;3:e170580. 10.1001/jamaoncol.2017.0580 PMC5824235 28494052

[5] Hunger SP (2018). Integrated Risk Stratification Using Minimal Residual Disease and Sentinel Genetic Alterations in Pediatric Acute Lymphoblastic Leukemia. J Clin Oncol.

[6] Brüggemann M, Kotrova M (2017). Minimal residual disease in adult ALL: technical aspects and implications for correct clinical interpretation. Blood Adv.

[7] Chen B, Jiang L, Zhong ML, Li JF, Li BS, Peng LJ (2018). Identification of fusion genes and characterization of transcriptome features in T-cell acute lymphoblastic leukemia. Proc Natl Acad Sci U S A.

[8] Liu Y, Easton J, Shao Y, Maciaszek J, Wang Z, Wilkinson M (2017). The genomic landscape of pediatric and young adult T-lineage acute lymphoblastic leukemia. Nat Genet.

[9] Malyukova A, Dohda T, von der Lehr N, Akhoondi S, Corcoran M, Heyman M (2007). The tumor suppressor gene hCDC4 is frequently mutated in human T-cell acute lymphoblastic leukemia with functional consequences for Notch signaling. Cancer Res.

[10] Gonzalez-Garcia S, Garcia-Peydro M, Alcain J, Toribio ML (2012). Notch1 and IL-7 receptor signalling in early T-cell development and leukaemia. Curr Top Microbiol Immunol.

[11] Asnafi V, Buzyn A, Le Noir S, Baleydier F, Simon A, Beldjord K (2009). NOTCH1/FBXW7 mutation identifies a large subgroup with favorable outcome in adult T-cell acute lymphoblastic leukemia (T-ALL): a Group for Research on Adult Acute Lymphoblastic Leukemia (GRAALL) study. Blood.

[12] Trinquand A, Tanguy-Schmidt A, Ben Abdelali R, Lambert J, Beldjord K, Lengliné E (2013). Toward a NOTCH1/FBXW7/RAS/PTEN-based oncogenetic risk classification of adult T-cell acute lymphoblastic leukemia: a Group for Research in Adult Acute Lymphoblastic Leukemia study. J Clin Oncol.

[13] Baldus CD, Thibaut J, Goekbuget N, Stroux A, Schlee C, Mossner M (2009). Prognostic implications of NOTCH1 and FBXW7 mutations in adult acute T-lymphoblastic leukemia. Haematologica.

[14] Mansour MR, Sulis ML, Duke V, Foroni L, Jenkinson S, Koo K (2009). Prognostic implications of NOTCH1 and FBXW7 mutations in adults with T-cell acute lymphoblastic leukemia treated on the MRC UKALLXII/ECOG E2993 protocol. J Clin Oncol.

[15] Rousseaux S, Debernardi A, Jacquiau B, Vitte AL, Vesin A, Nagy-Mignotte H et al. Ectopic activation of germline and placental genes identifies aggressive metastasis-prone lung cancers. Sci Transl Med. 2013;5:186ra66. 10.1126/scitranslmed.3005723 PMC4818008 23698379

[16] Rousseaux S, Wang J, Khochbin S (2013). Cancer hallmarks sustained by ectopic activations of placenta/male germline genes. Cell Cycle.

[17] Maxfield KE, Taus PJ, Corcoran K, Wooten J, Macion J, Zhou Y (2015). Comprehensive functional characterization of cancer-testis antigens defines obligate participation in multiple hallmarks of cancer. Nat Commun.

[18] Wang C, Gu Y, Zhang K, Xie K, Zhu M, Dai N (2016). Systematic identification of genes with a cancer-testis expression pattern in 19 cancer types. Nat Commun.

[19] Gordeeva O (2018). Cancer-testis antigens: Unique cancer stem cell biomarkers and targets for cancer therapy. Semin Cancer Biol.

[20] Le Bescont A, Vitte AL, Debernardi A, Curtet S, Buchou T, Vayr J (2015). Receptor-Independent Ectopic Activity of Prolactin Predicts Aggressive Lung Tumors and Indicates HDACi-Based Therapeutic Strategies. Antioxid Redox Signal.

[21] Wang J, Rousseaux S, Khochbin S (2014). Sustaining cancer through addictive ectopic gene activation. Curr Opin Oncol.

[22] Wang J, Mi JQ, Debernardi A, Vitte AL, Emadali A, Meyer JA (2015). A six gene expression signature defines aggressive subtypes and predicts outcome in childhood and adult acute lymphoblastic leukemia. Oncotarget.

[23] Emadali A, Rousseaux S, Bruder-Costa J, Rome C, Duley S, Hamaidia S (2013). Identification of a novel BET bromodomain inhibitor-sensitive, gene regulatory circuit that controls Rituximab response and tumour growth in aggressive lymphoid cancers. EMBO Mol Med.

[24] El Kennani S, Adrait A, Permiakova O, Hesse AM, Ialy-Radio C, Ferro M (2018). Systematic quantitative analysis of H2A and H2B variants by targeted proteomics. Epigenetics Chromatin.

[25] Tanaka-Hayashi A, Hayashi S, Inoue R, Ito T, Konno K, Yoshida T (2015). Is D-aspartate produced by glutamic-oxaloacetic transaminase-1 like 1 (Got1l1): a putative aspartate racemase?. Amino Acids.

[26] Li L, Zeng Q, Bhutkar A, Galvan JA, Karamitopoulou E, Noordermeer D (2018). GKAP Acts as a Genetic Modulator of NMDAR Signaling to Govern Invasive Tumor Growth. Cancer Cell.

[27] Ma JB, Ye K, Patel DJ (2004). Structural basis for overhang-specific small interfering RNA recognition by the PAZ domain. Nature.

[28] Wang Y, Xu Z, Jiang J, Xu C, Kang J, Xiao L, et al. Endogenous miRNA sponge lincRNA-RoR regulates Oct4, Nanog, and Sox2 in human embryonic stem cell self-renewal. Dev Cell. 2013;25:69–80. 10.1016/j.devcel.2013.03.002 23541921

[29] Katsushima K, Natsume A, Ohka F, Shinjo K, Hatanaka A, Ichimura N (2016). Targeting the Notch-regulated non-coding RNA TUG1 for glioma treatment. Nat Commun.

[30] Signer RA, Magee JA, Salic A, Morrison SJ (2014). Haematopoietic stem cells require a highly regulated protein synthesis rate. Nature.

[31] Blanco S, Bandiera R, Popis M, Hussain S, Lombard P, Aleksic J (2016). Stem cell function and stress response are controlled by protein synthesis. Nature.

[32] Cai X, Gao L, Teng L, Ge J, Oo ZM, Kumar AR (2015). Runx1 Deficiency Decreases Ribosome Biogenesis and Confers Stress Resistance to Hematopoietic Stem and Progenitor Cells. Cell Stem Cell.

[33] Mitchison TJ (2012). The proliferation rate paradox in antimitotic chemotherapy. Mol Biol Cell.

[34] Orth JD, Kohler RH, Foijer F, Sorger PK, Weissleder R, Mitchison TJ (2011). Analysis of mitosis and antimitotic drug responses in tumors by in vivo microscopy and single-cell pharmacodynamics. Cancer Res.

[35] Weinberg RA (1995). The retinoblastoma protein and cell cycle control. Cell.

[36] Nevins JR, Leone G, DeGregori J, Jakoi L (1997). Role of the Rb/E2F pathway in cell growth control. J Cell Physiol.

[37] Harbour JW, Dean DC (2000). The Rb/E2F pathway: expanding roles and emerging paradigms. Genes Dev.

[38] Kwon JS, Everetts NJ, Wang X, Wang W, Della Croce K, Xing J (2017). Controlling Depth of Cellular Quiescence by an Rb-E2F Network Switch. Cell Rep.

[39] Flotho C, Coustan-Smith E, Pei D, Cheng C, Song G, Pui CH (2007). A set of genes that regulate cell proliferation predicts treatment outcome in childhood acute lymphoblastic leukemia. Blood.

[40] Vannini N, Girotra M, Naveiras O, Nikitin G, Campos V, Giger S (2016). Specification of haematopoietic stem cell fate via modulation of mitochondrial activity. Nat Commun.

[41] Khacho M, Clark A, Svoboda DS, Azzi J, MacLaurin JG, Meghaizel C (2016). Mitochondrial Dynamics Impacts Stem Cell Identity and Fate Decisions by Regulating a Nuclear Transcriptional Program. Cell Stem Cell.

[42] Mi JQ, Wang X, Yao Y, Lu HJ, Jiang XX, Zhou JF (2012). Newly diagnosed acute lymphoblastic leukemia in China (II): prognosis related to genetic abnormalities in a series of 1091 cases. Leukemia.

[43] Chen B, Wang YY, Shen Y, Zhang WN, He HY, Zhu YM (2012). Newly diagnosed acute lymphoblastic leukemia in China (I): abnormal genetic patterns in 1346 childhood and adult cases and their comparison with the reports from Western countries. Leukemia.

[44] Zuurbier L, Petricoin EF, Vuerhard MJ, Calvert V, Kooi C, Buijs-Gladdines JG (2012). The significance of PTEN and AKT aberrations in pediatric T-cell acute lymphoblastic leukemia. Haematologica.

[45] Subramanian A, Tamayo P, Mootha VK, Mukherjee S, Ebert BL, Gillette MA (2005). Gene set enrichment analysis: a knowledge-based approach for interpreting genome-wide expression profiles. Proc Natl Acad Sci U S A.

[46] Mootha VK, Lindgren CM, Eriksson KF, Subramanian A, Sihag S, Lehar J (2003). PGC-1alpha-responsive genes involved in oxidative phosphorylation are coordinately downregulated in human diabetes. Nat Genet.

