# Supplementary material for: Ectopic expression of a combination of 5 genes detects high risk forms of T-cell acute lymphoblastic leukemia
Source: BMC Genomics. 2022 Jun 24;23:467. doi: 10.1186/s12864-022-08688-1 (PMC9233359; doi:10.1186/s12864-022-08688-1)
Supplement: Supplementary file 1 — Additional file 1. [file 12864_2022_8688_MOESM1_ESM.docx]

**Supplementary Methods**

**Detection of genes with a tissue-restricted expression profile**

To identify tissue-restricted genes, we used RNA-seq data of normal tissues provided by NCBI Sequence Read Archive (SRA) datasets PRJNA280600, PRJEB4337, PRJEB2445, PRJNA270632, PRJNA310976, NCBI GEO datasets GSE70741, GSE53096, ArrayExpress dataset E-MTAB-2836 and GTEx Portal. In total, we used genome-wide RNA-Seq data from 2955 samples corresponding to 48 different normal tissues. The input data are log-transformed RPKM values averaged by tissue and stage (adult, fetal, embryonic), in normal samples.

*Identification of tissue-predominant genes*

In the first step, we identify genes with a predominant expression in one or several tissues. Mean log-transformed RPKM values are calculated for each tissue and stage (based on the values of the corresponding samples), a distribution of mean values is obtained for each gene, and the predominant tissues are the outliers of this distribution of mean values.

In our approach, the outlier detection technique is based on the Z-score.

$$Zscore=\frac{x-mean}{std}$$

where $x$ corresponds to the mean log-transformed RPKM measured for one tissue group. If the Z-score of a tissue is above the threshold, this tissue is considered as predominant for the selected gene. We use an adaptive threshold for the Z-score, which depends on the total number of tissues according to the following empiric formula:

$$Zscorethreshold=0.6\frac{(N-1)}{\sqrt{N}}$$

where N is the total number of tissues.

For all genes with a predominant expression, we calculated the expression value for each predominant tissue (i.e. signal level), the maximum noise level and the signal to noise ratio by dividing the signal level by the maximum noise level.

*Identification of tissue-restricted genes*

In the second step, tissue-restricted genes were selected from tissue-predominant genes on the basis of simultaneously satisfying the following criteria: signal to noise ratio ≥ 5, signal level ≥ 0.5 and maximum noise level ≤ 0.3. The cutoff values were empirically defined by examining the expression profiles of some tissues-restricted genes which had already been identified as “tissue-restricted” in our previous work [15]*.*

By using RNA-Seq expression data in different normal human tissues, we identified 3195 transcripts whose expression was found restricted to testis, placenta or embryonic stem cells. Importantly, these genes are not expressed in normal hematopoietic tissues.

**Detection of ectopically expressed genes in T-ALL samples**

For each tissue-restricted gene, we established a threshold of log-transformed RPKM values differentiating background noise from expression. We determined this threshold by calculating the mean expression of the gene in 13 hematopoietic normal samples and adding 3 times the standard deviation to the mean. We then compared the expression value of each tumoral T-ALL sample with the threshold. If the expression value in a tumoral sample was above the threshold, then we considered the gene to be ectopically expressed in this sample. This strategy enabled us to binarize the expression values of these tissue-restricted genes in all our T-ALL RNA-seq data, and for each gene, a frequency of expression in T-ALL could be calculated.

**Association between ectopic expression and prognosis**

Considering each gene ectopically expressed in at least 10% of the T-ALL, we compared the survival between the group of T-ALL expressing the gene and the group of T-ALL not expressing the gene. Cox proportional hazard model was used in order to test if the expression of the gene was significantly associated with survival, successively considering overall (OS) and event-free survivals (EFS). The ectopic expression of a gene was considered as significantly associated with the survival if the Cox model p-value was less than 0.05 and the hazard ratio above 1.5. As a result of this approach, 18 genes were found associated with a lower OS and/or EFS probability.

The patients were also stratified by combining the expression status of the 18 genes (“expressed” versus “not expressed”), which also predicted prognosis. For instance, each of the T-ALL patients was assigned to one of three groups with respect to the total number of ectopically expressed genes: P1 where none of 18 genes was expressed, P2 with one or two genes expressed and P3 with more than three genes expressed. Survival prognosis was significantly associated with the total number of ectopically expressed genes (*p* < 10^-8^ for OS and *p* < 10^-9^ for EFS).

**Definition of optimal subsets of genes**

The 18 genes can be used as biomarkers to predict patient prognosis. Nevertheless, from a practical point of view, in order to develop a robust and easy-to-use prognostic test, it is also interesting to identify a smaller subset among the 18 genes that would efficiently stratify the patients according to their survival probability. More specifically, we designed a strategy aiming at identifying subsets of genes that would efficiently assign patients with the longest survival probability to the P1 group.

For this purpose, we tested all possible combinations of the 18 genes for their ability to differentiate T-ALL patients with different prognostic. To explore the impact of combined subsets of genes on survival, we calculated Kaplan-Meier survival estimates for all possible combinations. The total number of combinations is equal to 2^18^ -1 = 262 143 subsets. We obtained several subsets that maximize survival estimate in P1 group with a relatively small number of genes. Supp. Fig. S3 shows the efficiency of different subsets of genes depending on their size. For each subset size (from 1 gene to 18 genes) we calculated, for each of the possible combinations of genes, the survival probability of the P1 group at 60 months, and selected the combination with the highest survival probability. According to our results, at least five genes are necessary to efficiently stratify patient’s prognosis. Below each bar plot of Supp. Fig. S3 is the list of the top 5 of the optimized subsets of genes for each size. Our data show that these lists are identical for OS and EFS.

The TOP 5 of the subsets of genes optimized for the stratification of global T-ALL group that maximize survival (they were identical for OS and EFS):

- *5 genes*: ACTRT2, GOT1L1, SPATA45, TOPAZ1, ZPBP
- *6 genes*: ACTRT2, GOT1L1, GPR32, SPATA45, TOPAZ1, ZPBP
- *7 genes*: ACTRT2, GOT1L1, GPR32, SPATA45, TOPAZ1, TSBP1, ZPBP
- *8 genes*: ACTRT2, FLJ40194, GOT1L1, GPR32, SPATA45, TOPAZ1, TSBP1, ZPBP
- *9 genes*: ACTRT2, FLJ40194, GOT1L1, GPR32, SPATA45, TOPAZ1, TSBP1, ZDHHC22, ZPBP

In this study, we used the first optimal subset composed of five genes ACTRT2, GOT1L1, SPATA45, TOPAZ1 and ZPBP, significantly associated with prognosis (*p* < 10^-4^ for OS and *p* < 10^-5^ for EFS). Other subsets have similar properties.

**Supp. Figures and legends**

**Supp. Figure S1.** **O****n****c****ogenetic status, age and survival probabilities in adult T-ALL patients.** The left panels show pie charts with the distributions of N/F (A) NFRP (B) mutational status and age (C) amongst 86 T-ALL adult patients**.** The right panels are Kaplan–Meier curves comparing overall survival (OS) and event-free survival (EFS) of T-ALL adult patients according to their N/F (A) or NFRP (B) mutational status. **(C)** Kaplan-Meyer curves comparing the survival between patients below and above a threshold of 35 years old.

**
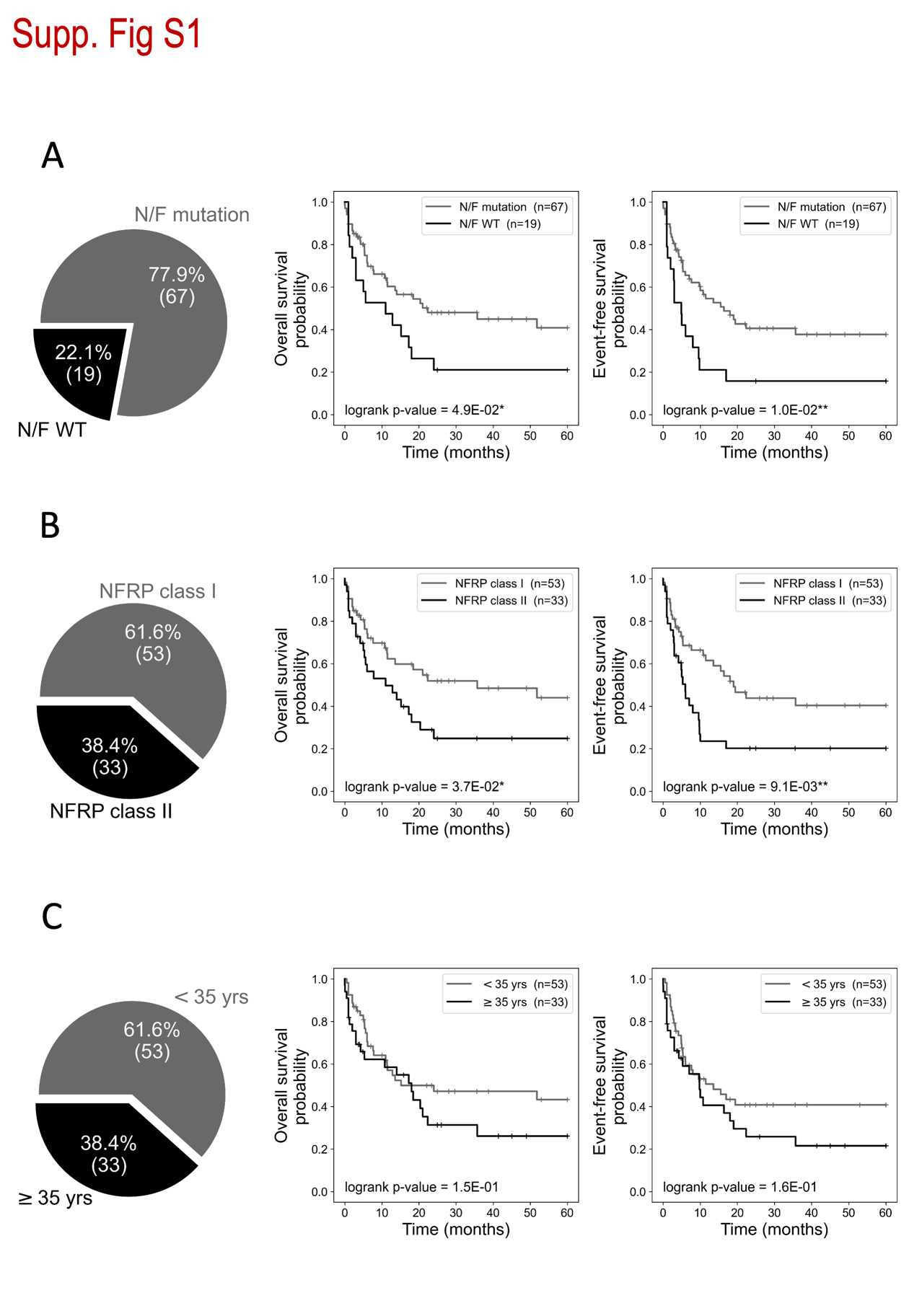
**

**Supp. Figure S2. Kaplan-Meyer survival curves corresponding to each of the 18 genes** comparing the overall (A) and event-free (B) univariate survival probabilities between the patients whose T-ALL expressed the gene (red line) and those whose leukemic cells did not (blue line).

**
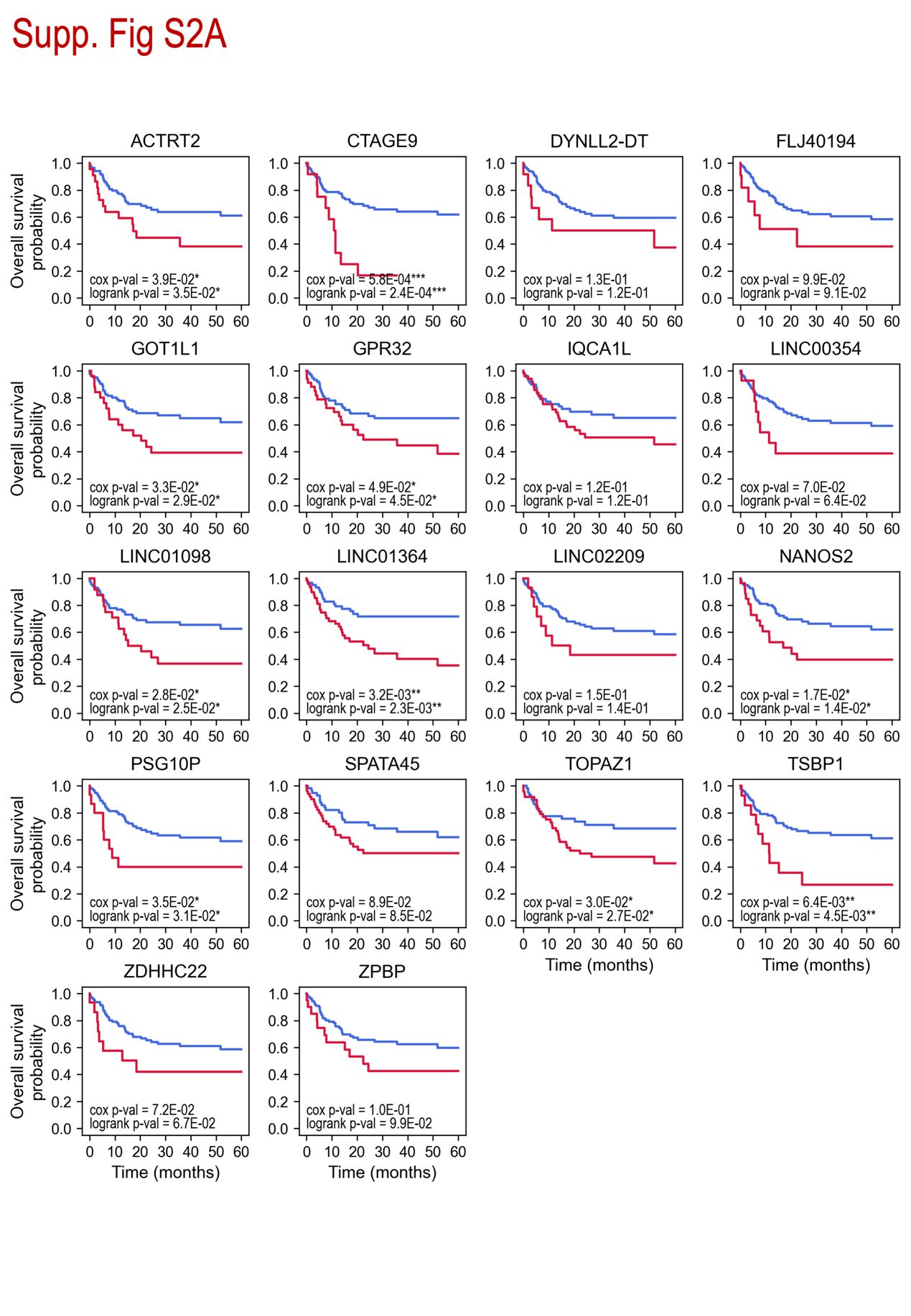
**

**
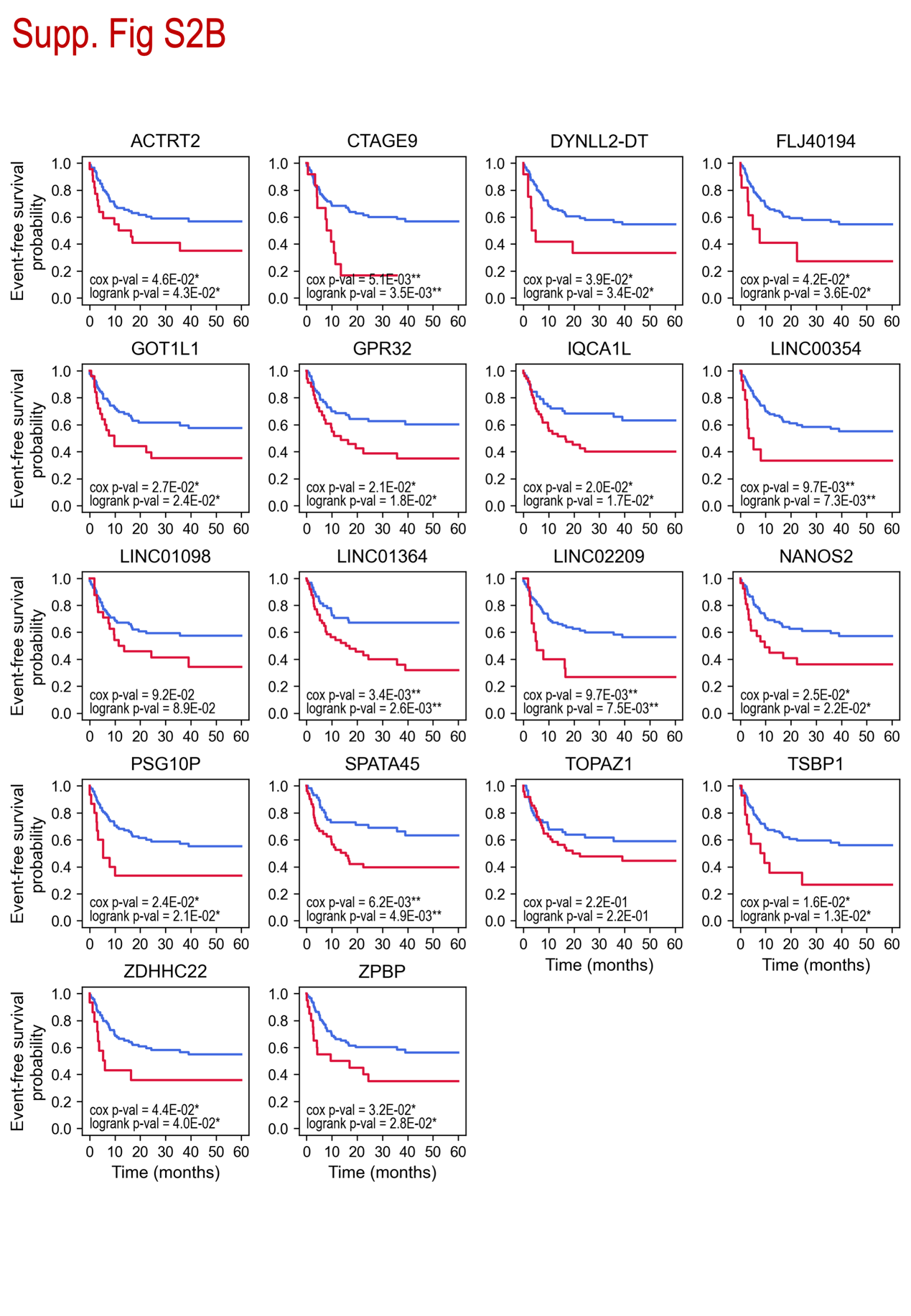
**

**Supp. Figure S3. Best overall survival probability (A) and event-free survival probability (B) in the 5-GEC prognosis group P1 at 60 months** obtained for subsets of genes of different sizes. A combination of at least 5 genes is necessary to obtain 100% survival probability of P1 in at least one subset. The top subsets of respectively 5, 6, 7, 8 and 9 genes allowing to attain a survival probability of 100% for P1 patients are shown in blue. Below each bar plot is the list of the TOP 5 of the optimized subsets of genes for each size. Our data show that these lists were all identical for OS and EFS.

**
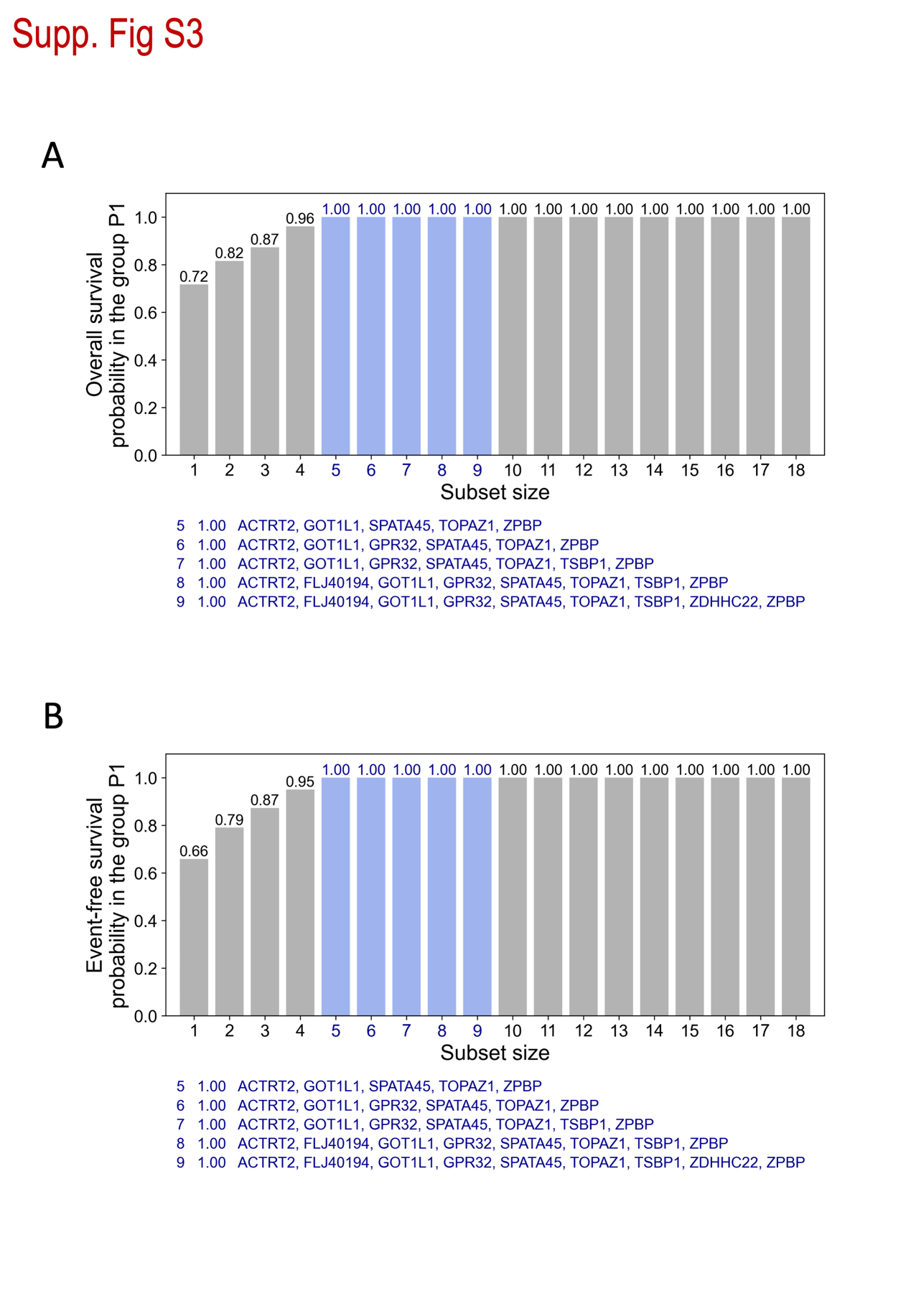
**

**Supp. Figure S4. Survival analysis of our test dataset with the 5-GEC classifying tool in combination with MRD. (**A) Left panel: pie chart showing the distribution of MRD negative (grey) or positive (black) patients in our test dataset (n=29, MRD status was missing for three patients). Center and right panels: Kaplan-Meier curves comparing overall survival (OS, center panel) and event-free survival (EFS, right panel) between MRD negative (n=13) and positive (n=16) T-ALL adult patients; (B) Bar plots showing the respective proportions of 5-GEC negative (blue) or positive (grey) T-ALL according to MRD status (as indicated) in our test dataset. Although patients with positive MRD status are over-represented in the 5-GEC positive group, this relative enrichment is not significant (Fisher p-value = 0.364); (C) Kaplan-Meier curves comparing overall survival (OS, left panel) and event-free survival (EFS, right panel) between 5-GEC negative and 5-GEC positive T-ALL in adult patients of the test dataset considering patients with negative MRD status (total n=13).

**
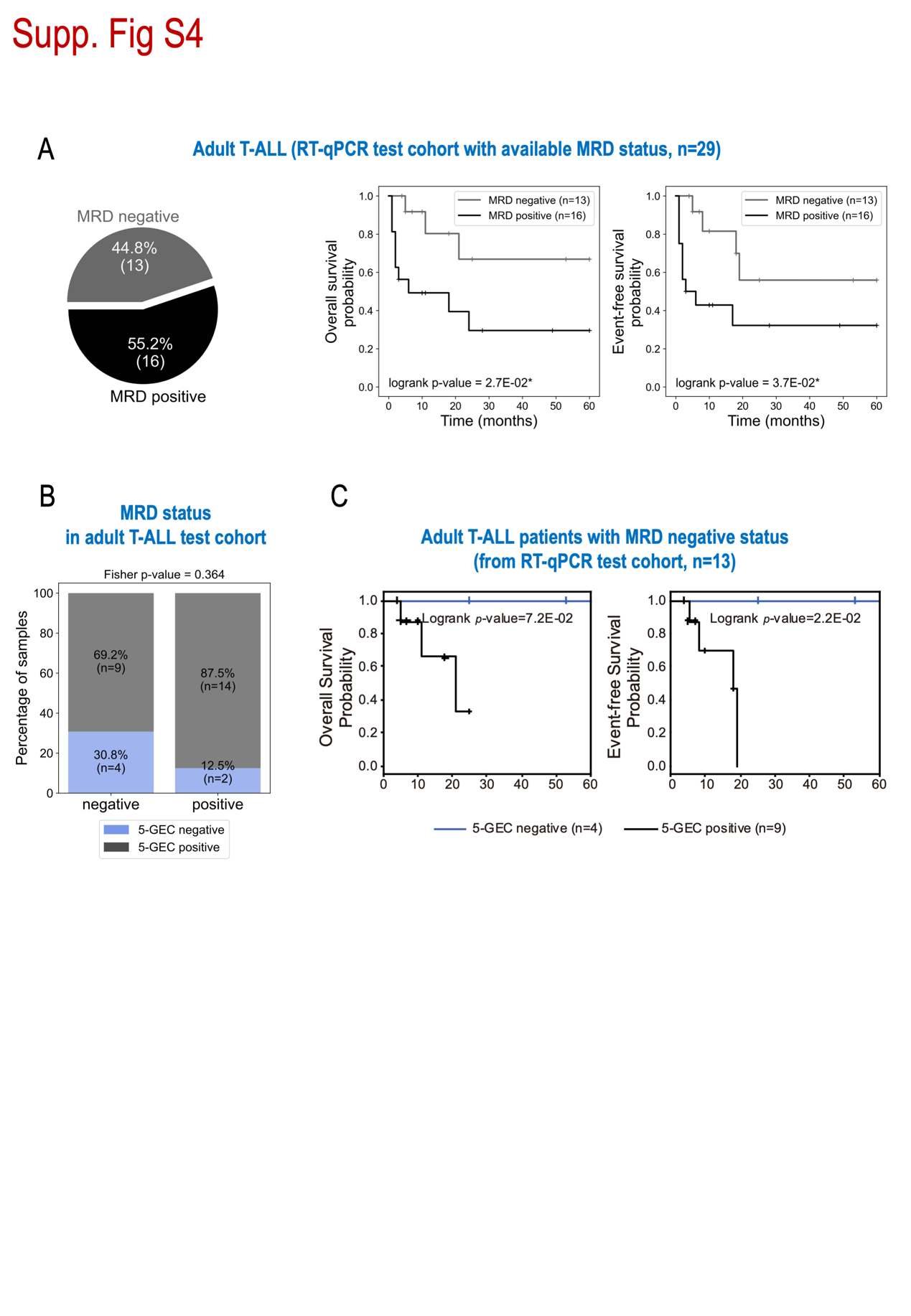
**

**Supp. Figure S5. Volcano plots (left panels) and heatmaps (right panels) illustrating the transcriptomic profiles** of 5-GEC positive versus negative T-ALL (A) or MRD positive versus negative T-ALL (B) considering all patients of the training set (adult and children, n = 109). The p-values used in volcano plots were calculated with the Wilcoxon statistical test. The differentially expressed genes used for the heatmaps were selected with a p-value < 0.05 and abs (ratio) > 1.5.


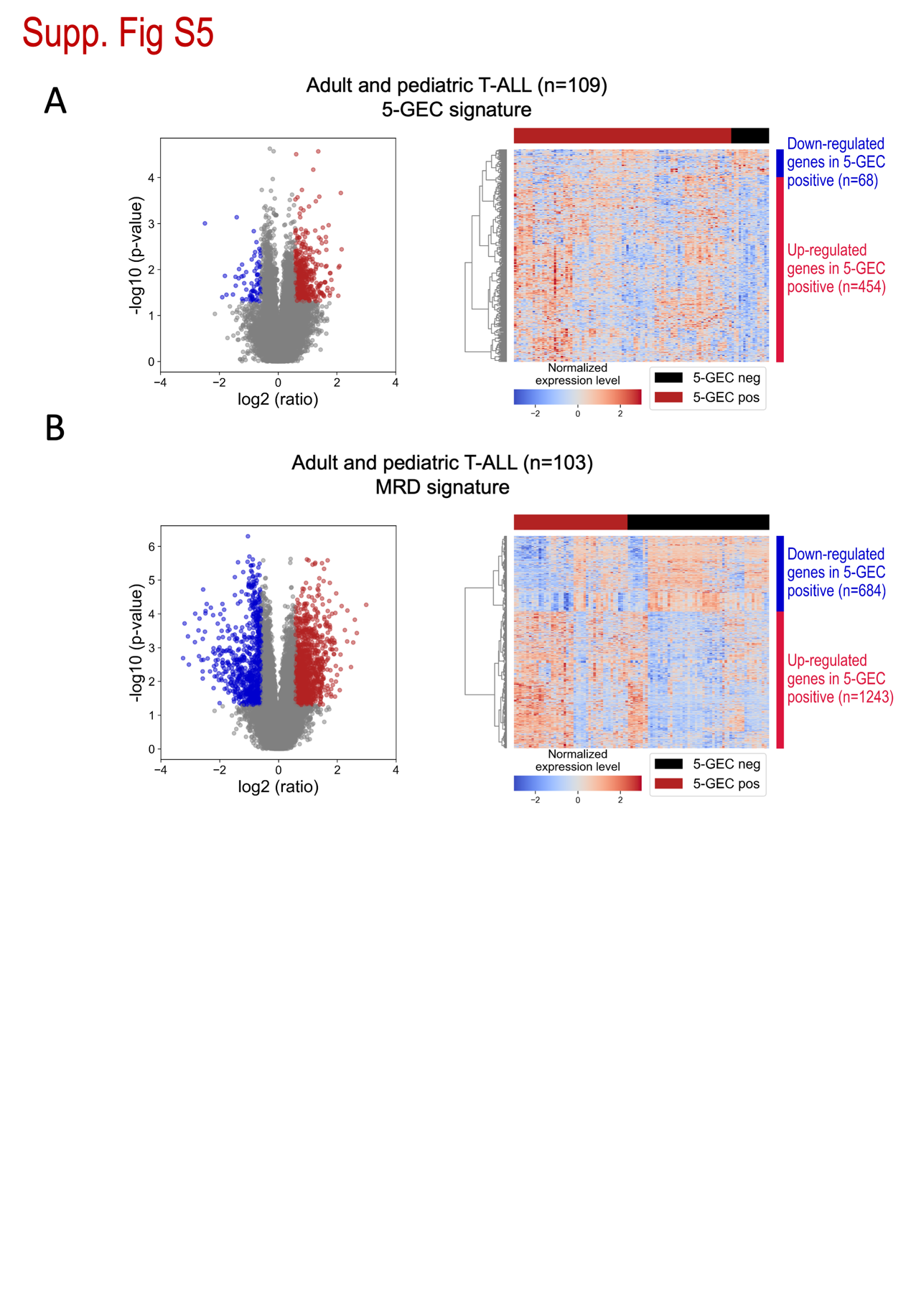


**Supp. Figure S6**. **Heatmap with hierarchical clustering of NES values from Gene Set Enrichment Analysis (GSEA)** summarizing specific characteristics (genesets and pathways) of the transcriptomic signatures which are shared or different between 5-GEC positive T-ALL, considering adult patients or children, or with the signature of MRD positive T-ALL, as indicated**.**

**
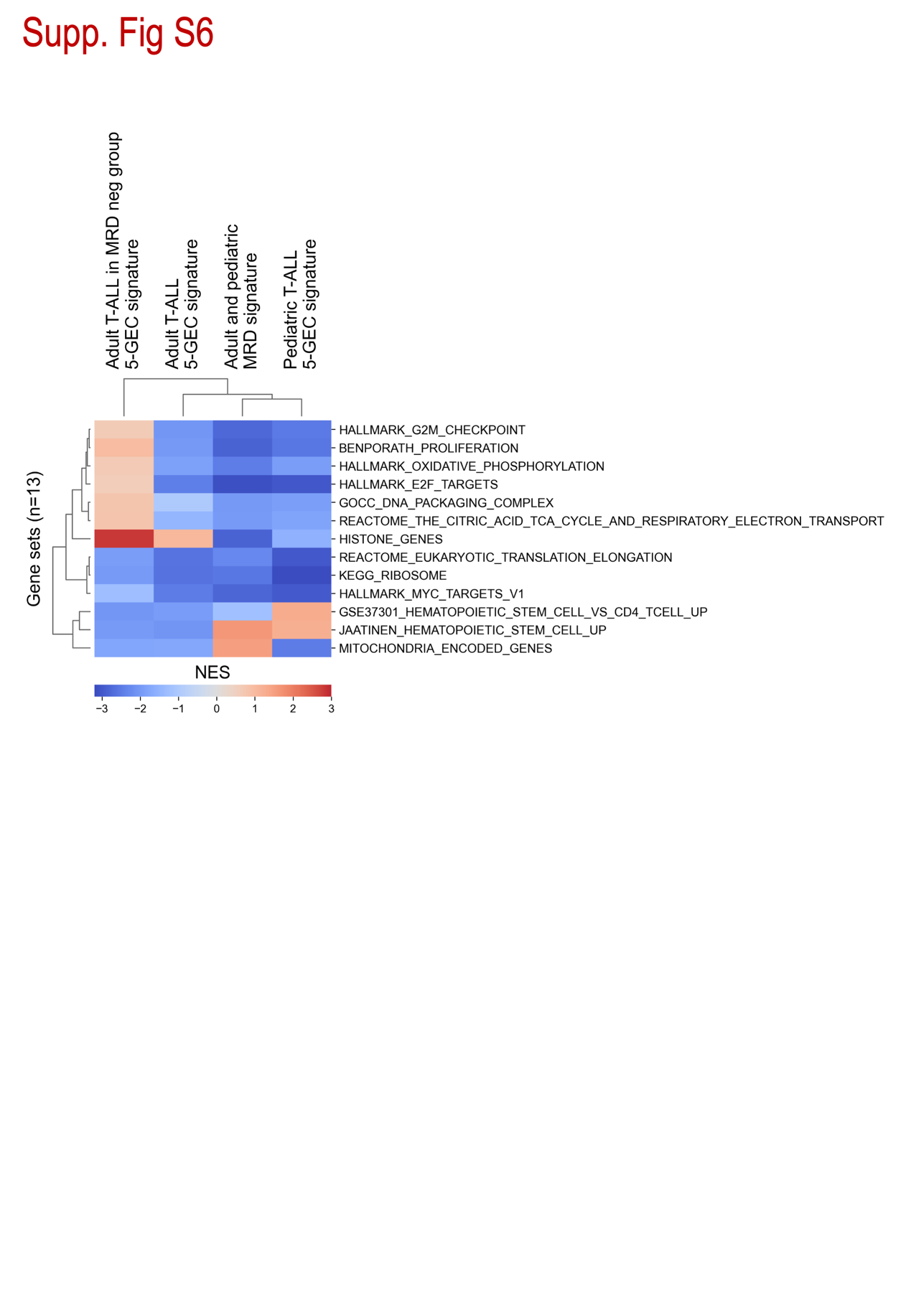
**

**Supp. Figure S7**. **Gene Set Enrichment Analysis (GSEA) showing the enrichment or depletion of specific genesets characterizing the transcriptomic signatures which are shared or different between 5-GEC positive T-ALL, considering adult patients or children, or with the signature of MRD positive T-ALL.**

The geneset “histone genes” corresponds to the human histone encoding genes identified in El Kennani et al 2018. The geneset “mitochondria encoded genes” is constituted of the 13 mitochondrial genes of the human genome. neg: negative; pos: positive. All other genesets were selected from the MSIG database of the Broad Institute (categories C2, C5, C7 or H of the MsigDB). neg: negative; pos: positive.


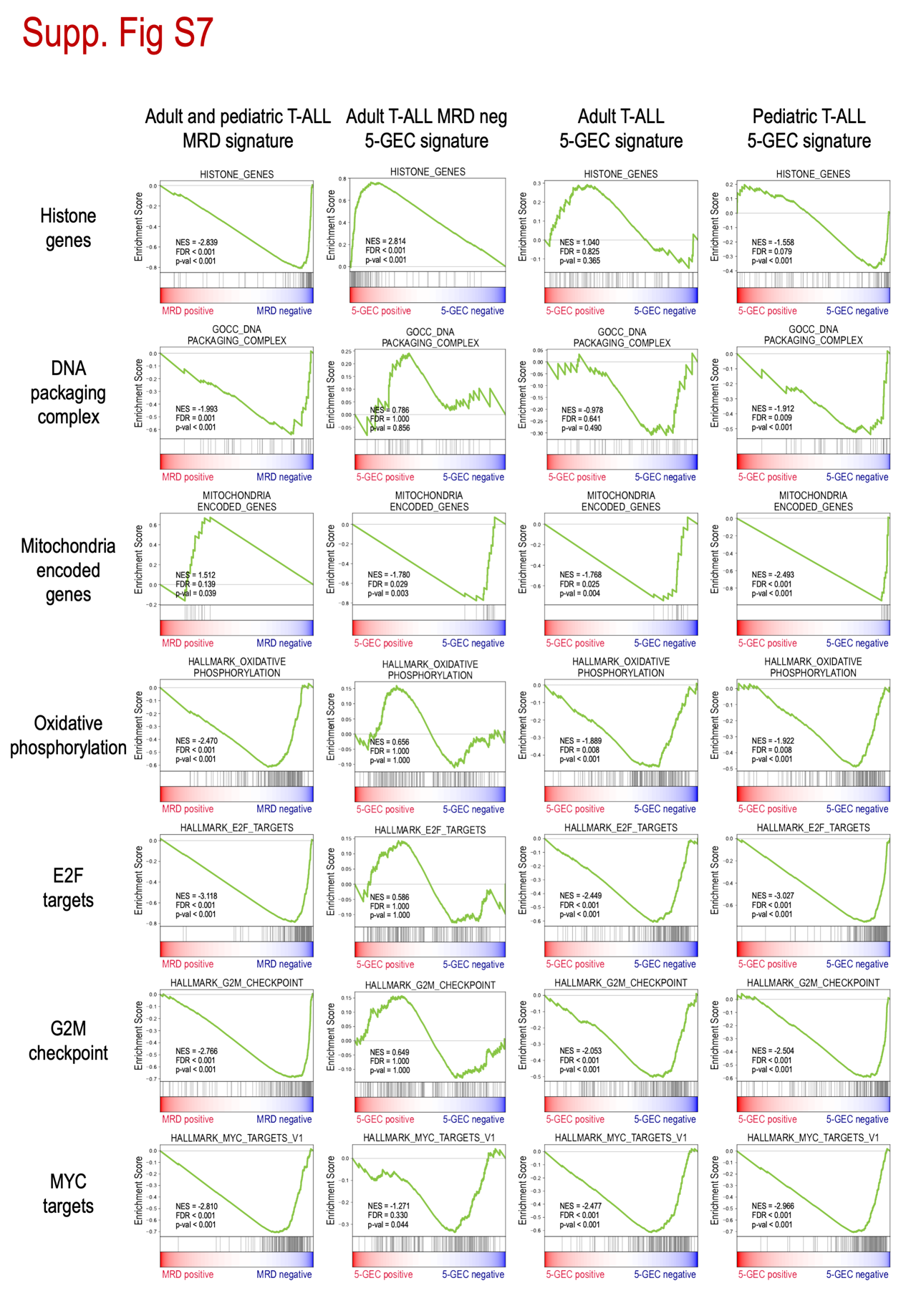


**Legends and supplemental tables S1 to S5**

**Table S1**. **Description of the clinical and molecular data for each of the three cohorts of the present study**, including T-ALL adult patients with RNA-seq (n-54), or with RT-QPCR data (n=32), and T-ALL pediatric patients (n=55).

A. Overview and respective sizes of our three T-ALL cohorts;

B. Clinical and biological characteristics of our three T-ALL cohorts.


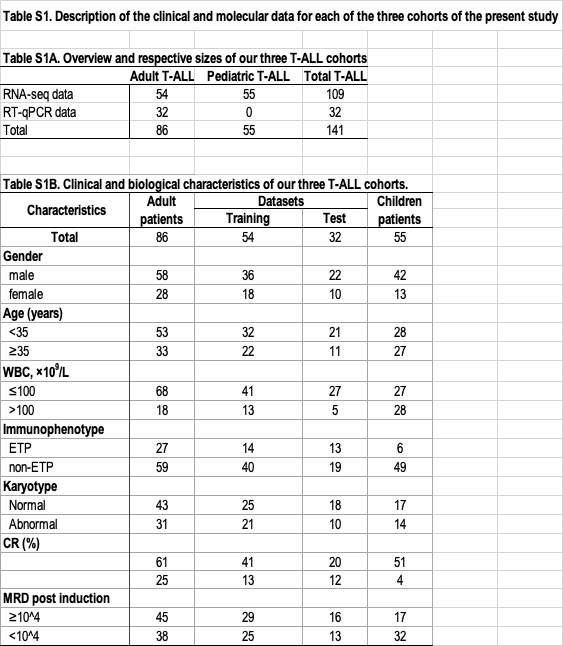


**Table S2**. List of 18 ectopically expressed genes in T-ALL samples and their association with prognosis in T-ALL of the training cohort (adult and children, n = 109).


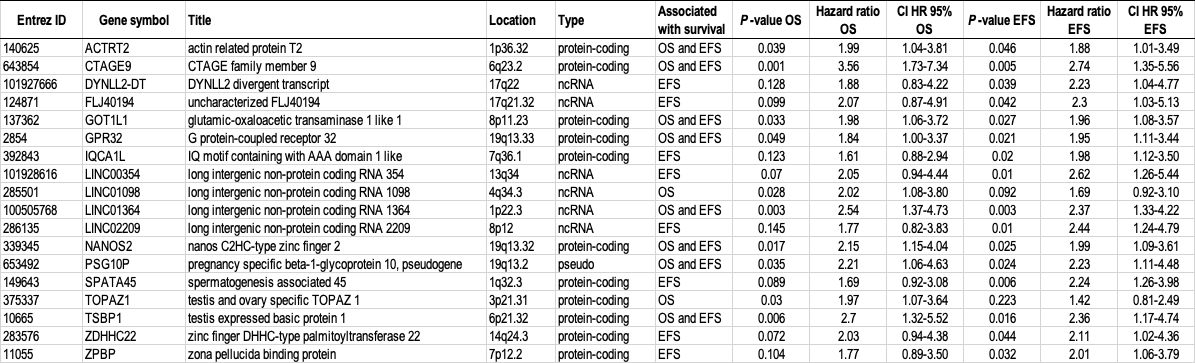


**Table S3**. Multivariate Cox model evaluating the probabilities of survival according to the ON/OFF status of the selected 18 genes.


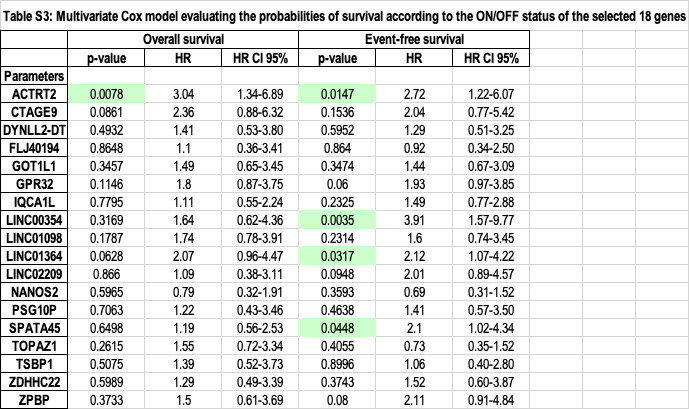


**Table S4**. Multivariate survival analysis combining 5-GEC and age.


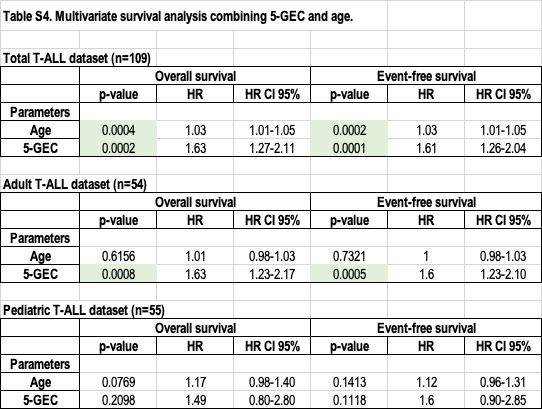


**Table S5. Most significantly enriched or depleted genesets resulting from the GSEA analyses of the five following transcriptomic signatures** including i/ 5-GEC signature (positive versus negative) in all T-ALL (pediatric and adult, n=109), ii/ 5-GEC signature in adult T-ALL (adult samples of the training cohort, n=54), iii/ 5-GEC signature in adult T-ALL with MRD negative status (subset of the adult training cohort, n=25), iv/ 5-GEC signature in pediatric T-ALL (children of the training cohort, n=55), v/ MRD signature (positive versus negative) in all T-ALL (pediatric and adult samples of the training cohort for whom the MRD status is available, n=103).

Please see excel file entitled “peng_R1_main_Table_1_clin_oncogenetic_data_adult_TALL”.
